# Postoperative Bowel Perforation due to Heterotopic Ossification (Myositis Ossificans Traumatica): A Case Report and Review of the Literature

**DOI:** 10.1155/2011/908514

**Published:** 2011-07-10

**Authors:** Victoria Valinluck Lao, Oliver B. Lao, Edgar Figueredo

**Affiliations:** Department of Surgery, University of Washington, Seattle, WA 98195, USA

## Abstract

Heterotopic ossification (HO) is the ectopic development of normal bone within soft tissue that can occur after traumatic injury. It is uncommon and may be missed or misdiagnosed, which can lead to complications. We report the case of an 84-year-old male with a previous history of a laparotomy who underwent resection of an intra-abdominal tumor through a midline incision. On postoperative day six, the patient was taken to the operating room, as succus was draining from the incision. Upon re-exploration, sharp bone-like material was found in the wound directly adjacent to an enterotomy. Pathology confirmed mature lamellar bone and the diagnosis of HO. This is the first report of postoperative intestinal perforation secondary to HO in a midline wound. We report this case to encourage accurate reporting of HO and its morbidity and complications for the benefit of appropriate surgical planning and epidemiologic tracking of outcomes.

## 1. Background

Heterotopic ossification is the ectopic development of normal bone within soft tissue. In the setting of traumatic injury, such as surgery, it may be referred to as myositis ossificans traumatica and carries the eponyms of Rider's bones and Shooter's bones when found in the adductor muscles and deltoid muscles, respectively. The underlying etiology is unknown but commonly occurs following operations, central nervous system injury, musculoskeletal injuries, burns, vasculopathies, and arthropathies [1, 2]. Morbidity is largely a function of the anatomic location of the ossification. In the orthopedic literature, heterotopic ossification is significant for its role in causing disability in a joint, as it inhibits full range of motion. When found that in an abdominal scar, it may cause symptoms such as discomfort or pain, especially in active patients. When it occurs in dependent areas, it may cause tissue damage and skin breakdown. Aside from the physical discomfort, there is psychological discomfort related to the possibility of harboring malignancy or recurrence if the initial operation was for malignancy. Rarely, malignant degeneration to osteosarcoma has been reported [3, 4]. While rare, heterotopic ossification may occur in abdominal scars.

No true estimate of the incidence heterotopic ossification exists and underscoring is rarity, especially in the abdominal surgery cohort. Select case series of three, eleven, and twenty-three patients combined with isolated case reports provide the majority of our understanding of this condition [5–7], highlighting that heterotopic ossification can be recurrent and that it should not be misinterpreted for cancer. Given the scarcity of reports, descriptions of each incidence and of the management and morbidity is paramount for improved understanding, operative planning and tracking of outcomes. Here, we report the unique case of an 84-year-old patient who suffered postoperative intestinal perforation from heterotopic ossification in his midline abdominal wound, an occurrence that has not been previously described. 

## 2. Case Presentation

An 84-year-old man was worked up as an outpatient for tachycardia and hypertension. An abdominal CT scan demonstrated a para-aortic mass, suspicious for a neuroendocrine tumor. Biopsies were positive for synaptophysin and CD117, and the patient was initially treated with octreotide for a presumed carcinoid tumor. Further work-up revealed serum metanephrines to be twenty times normal levels. This along with subsequent episodes of hypertension, chest pain, electrocardiography changes and repeated non-ST elevation myocardial infarctions led to a change in diagnosis to pheochromocytoma or paraganglioma. The patient was appropriately resuscitated and started on a non-selective alpha-blocker, phenoxybenzamine, followed by a beta-blocker. 

The patient's medical history was significant for an abdominal operation two years prior for a sigmoid diverticular bleed. At that time, he had a midline abdominal incision, bowel resection, end colostomy, and a rectal pouch. In the ensuing two years, he found that his colostomy was malpositioned, located on a skin crease, and, therefore, he had difficulty with stoma hygiene. Under our care, the patient was taken operating room for an exploratory laparotomy, tumor resection, and stoma revision.

Upon abdominal exploration, lysis of adhesions resulted in several serosal tears that were repaired primarily. One large tear required a partial small bowel resection and primary hand-sewn anastomosis. The tumor was encountered adherent to the aorta and carefully resected. The end colostomy was resited, and careful exploration of the abdominal cavity ensured no missed enterotomies. The fascia was closed with two types of absorbable suture, and the skin was closed with staples. 

On postoperative day five, he was noted to have leakage of serosanguinous fluid from the midline wound. Some staples were removed from the skin, and the wound was packed. In the interim, the colostomy had regained function. On postoperative day six, succus was found to be draining from the inferior portion of the wound, and the patient was taken back to the operating room for a re-exploration with a pre-operative diagnosis of anastomotic leak or failure of enterotomy repair.

**Figure 1:**
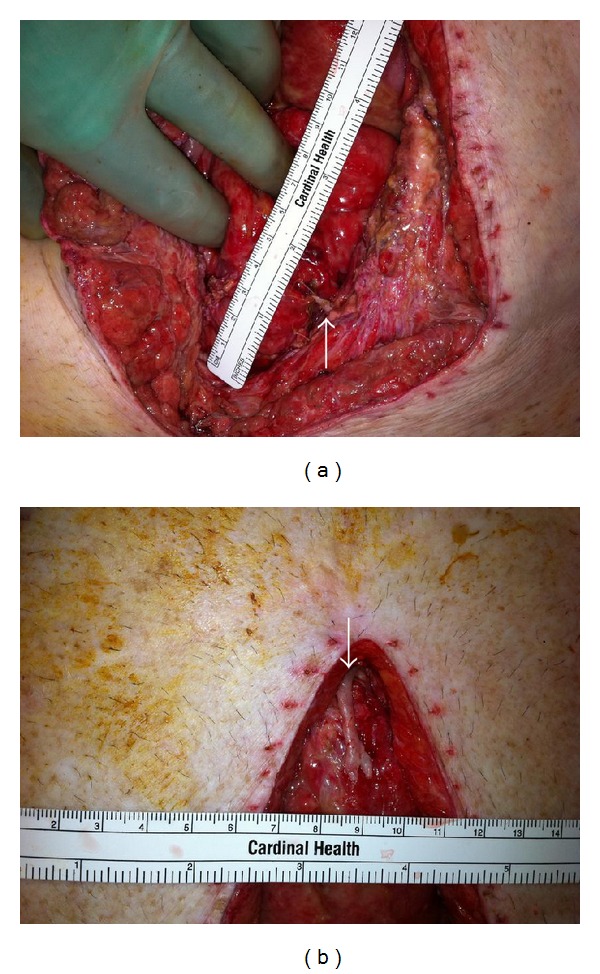
Intraoperative photographs demonstrating the (a) location of 3 mm × 1 cm heterotopic ossification within inferior midline wound and repaired small bowel perforation. Arrow denotes heterotopic ossification emanating from the wound. (b) Additional area of heterotopic ossification, 7 mm × 2.3 cm, in superior aspect of the wound. Arrow denotes heterotopic ossification emanating from the wound.

Upon re-exploration, the remaining staples were removed. The inferior portion of the midline wound was opened first, as it was draining succus. We quickly identified, immediately under the fascia, an enterotomy that was draining succus. This area was oversewn in two layers. The remainder of the wound was opened, and a thorough exploration of the abdominal cavity did not reveal any other evidence of leakage or perforation. All previous anastomosis and enterotomy repairs were intact. During that search, a hard 3 mm × 1 cm irregularly shaped lesion with a sharp point was encountered along the inferior portion of the midline wound directly adjacent to the noted enterotomy (Figure 1(a)). The remainder of the midline wound was explored, and a similarly hard, larger 2.3 cm × 7 mm lesion was found at the superior portion of the wound (Figure 1(b)). Both specimens had the consistency of bone or plastic. They were both removed and submitted to pathology. The wound was closed with a biosynthetic patch underlay. Subsequently, the patient recovered and regained enteral autonomy. 

**Figure 2:**
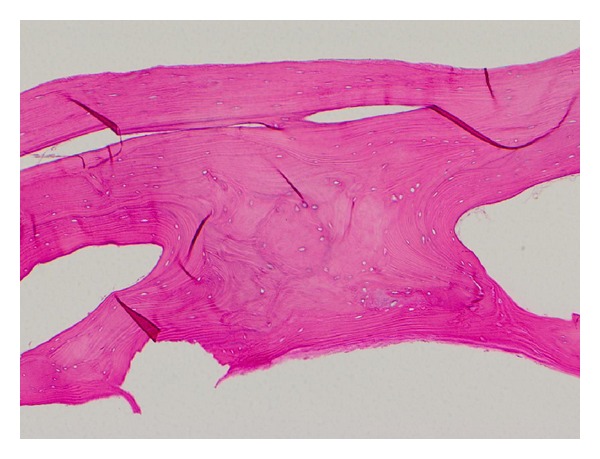
Histopathology of heterotopic ossification illustrating normal, mature lamellar bone.

**Figure 3:**
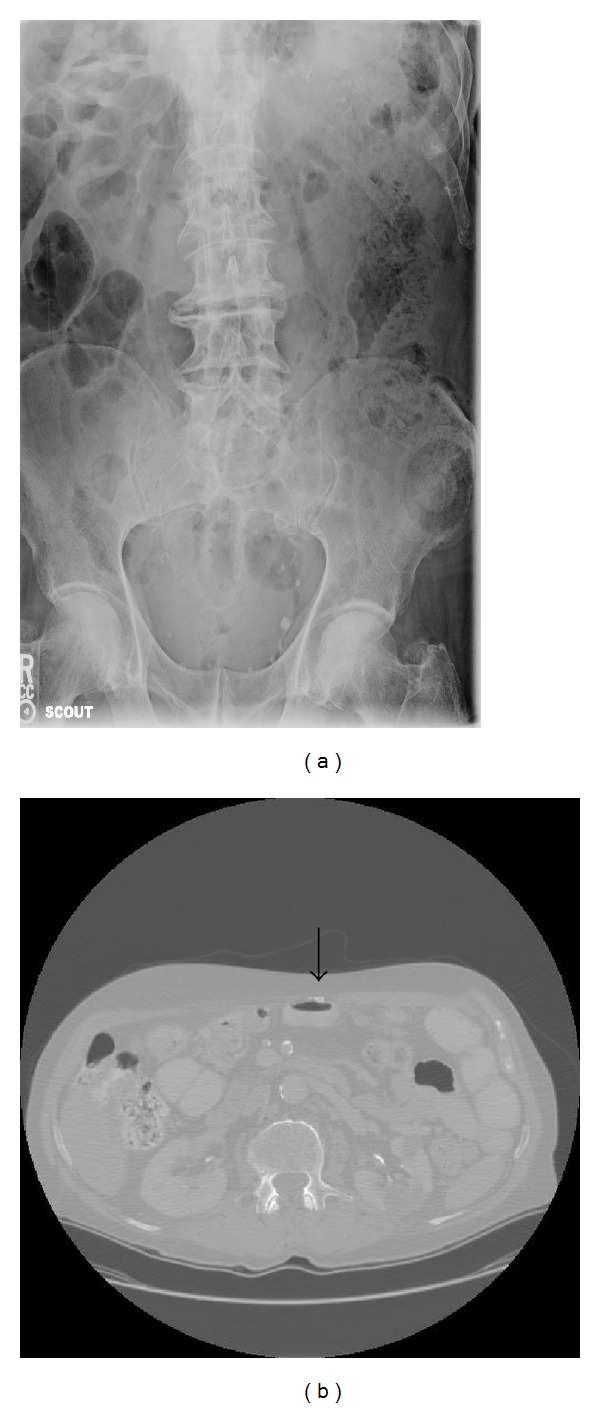
Preoperative abdominal showing (a) no evidence of heterotopic ossification on abdominal plain film. However, there is clear (b) evidence of heterotopic ossification in the lower midline abdominal scar adjacent to bowel wall seen on abdominal CT (computerized tomography) scan. Arrow denotes heterotopic ossification.

Microscopically, the two specimens were found to be normal, mature lamellar bone (Figure 2). There was no cellular atypia or evidence of malignancy. Rereview of the plain radiographs performed pre-operatively did not demonstrate any visible evidence of the heterotopic ossification; however, computerized tomography images performed pre-operatively demonstrated evidence of heterotopic ossification at the inferior portion of the wound in the area where the two lesions were found intraoperatively (Figure 3). Given these radiologic findings, we suspect that the heterotopic ossifications developed after his operation two years prior for perforated sigmoid diverticular bleeding, as opposed to developing within the fifth or sixth postoperative day from his more recent exploratory laparotomy, tumor resection, and stoma revision. 

## 3. Discussion and Conclusion

A thorough search of the literature revealed several reports regarding the presence and treatment of heterotopic ossification in a diversity of locations in association with a variety of traumatic injuries [4, 5, 7–16], but only one similar case of heterotopic ossification leading to intestinal perforation [17]. Our case is the first to report intestinal perforation from heterotopic ossification in the postoperative setting. 

Reports of heterotopic ossification specifically within abdominal wall can be found in the literature dating back to the 1940s [18] with a surge of reports in the 1970s [11–13, 15, 19–21]. Since that time, there have been subsequent reports with different inciting mechanisms and complications. Development of heterotopic ossification within the abdominal wall following penetrating abdominal trauma makes up the majority of reports, but development after blunt abdominal trauma has also been noted [10]. Heterotopic ossification has been described in conjunction with burns as well [14, 22, 23]. Various uncommon locations for heterotopic, such as the hand, head, kidney, and even the popliteal fossa have been reported [8, 9, 16, 24–26] in addition to the more commonly reported cases following orthopedic procedures such as total hip arthroplasties and open repair of acetabular fractures [2]. Penetrating trauma to the abdomen has been reported to cause ossification within the mesentery [27]. 

Focusing solely on abdominal heterotopic ossification, one of the earliest case series conducted in 1975 looked at heterotopic ossification in 23 abdominal incisions. They found a male preponderance (79% versus 21%) with an average age of 55. They noted the possibility of reabsorption and disappearance in some patients but urged removal if symptomatic and cautioned against mistaking it for a malignant lesion [13]. Male preponderance is further supported from subsequent case series in the gynecologic literature [7] along with a radiologic review where ten of the eleven cases were male [5]. A study based upon radiographic review showed a median age of 40 years-old (range 20–76 years) with a scar size of 2.2 cm (range 0–4.9 cm) and found the time from traumatic event to development of radiologic findings may be as early as eleven days (mean 6.8 months) [5]. The most recent series of 3 cases included patients with ages between 51 and 74 years old and found development of ossification between 2 and 4 months following the traumatic event [28]. All were treated with excision with the one recurrence receiving adjuvant radiotherapy of the excision site. Although the majority of these cases and series include adults, the pediatric population is not immune, as there are reports of heterotopic ossification in children as young as two years of age [29, 30].

There are two proposed mechanisms for the development of heterotopic ossification in abdominal wounds. The first is that heterotopic ossification develops from liberated bone fragments from the periosteum or perichondrium of either the xiphoid process or symphysis pubis that are deposited within and along an incision. This theory is supported by the fact that all reported cases have been associated with midline laparotomy wounds and none have been reported within transverse incisions [28]. The second theory is founded in the belief that the heterotopic ossification develops from immature pluripotent mesenchymal cells that are triggered by the inciting trauma to differentiate into either osteoblasts or chondroblasts [31]. A similar proposal has been made in the orthopedic literature. Kaplan et al. have suggested that four factors are necessary for the development of heterotopic ossification [6]:

1. an inciting event, such as trauma, but can be as trivial as a few torn muscle fibers,
2. an inductive signaling pathway, most probably secreted from injured cells in the form of protein,
3. supply of mesenchymal cells that are somewhat totipotent so that they differentiate into osteoblasts and chondroblasts under the appropriate signal,
4. an appropriate environment conducive to production of heterotopic bone.

Signals for osteoblast and chondroblast differentiation from mesenchymal cells may include bone morphogenetic protein-2 (BMP-2) as heterotopic ossification has been seen in patients receiving recombinant human bone morphogenetic protein-2 (rhBMP-2) for orthopedic procedures [32]. Although trauma is believed to be an underlying inciting factor, there may also be a genetic predisposition to the development of heterotopic ossification, as the presence of such ossification in a midline abdominal wound may indicate the possibility of similar deposits elsewhere in the body that have not previously been operated upon [31].

Regardless of the underlying etiology, management of a mass believed to be heterotopic ossification must take several factors into account. First, one must differentiate between benign heterotopic ossification and the possibility of a malignancy [13]. If the first operation was performed for cancer, one must exclude the possibility of scar recurrence following surgery for abdominal malignancy [33]. Excision or resection of the mass is usually indicated with symptoms (discomfort or pain) or to rule out malignancy. Following resection, the use of etidronate disodium may be useful in the prophylactic setting to help prevent recurrence [34], while radiotherapy may be used if and when those recurrences present themselves [28].

This case of postoperative intestinal perforation secondary to heterotopic ossification in a midline abdominal wound serves to demonstrate several important points. First, the preoperative evaluation and imaging of patients that have had previous operations should not only focus on the intra-abdominal pathology in question, but also include the possibility of heterotopic ossification in a previously operated wound. Second, close attention to the wound edges during fascial closure to ensure the absence of heterotopic ossification may prevent a similar occurrence. Finally, keeping an open mind when needing to reoperate in the immediate postoperative period will allow one to think of rare and remote possibilities such as the one we experienced instead of the more common (anastomotic leak). The surgeon should be alert to the possibility of heterotopic ossification, especially within a previously operated wound and be prepared for the difficulties it may cause during re-opening of an incision, during the operation itself, at closure of the wound and, if not removed, also in the postoperative setting. 

## 4. Consent

An institutional IRB was obtained for publication of this case report along with accompanying images. A copy of the documentation is available for review by the Editor of this journal upon request.
